# Migratory Foreign Bodies in the Aerodigestive Tract: The Importance of CT Imaging

**DOI:** 10.7759/cureus.21595

**Published:** 2022-01-25

**Authors:** Syed Zohaib Maroof Hussain, AMRUTHA KK, Abdul Wadood Mohammad, Muryum Khan

**Affiliations:** 1 ENT, Norfolk and Norwich University Hospital, Norwich, GBR; 2 Otolaryngology, KIMS Alshifa Super Speciality Hospital, Perinthalmanna, IND; 3 Otolaryngology - Head and Neck Surgery, Norfolk and Norwich University Hospitals, Norwich, GBR; 4 Medicine, Queen Elizabeth Hospital King's Lynn, King's Lynn, GBR

**Keywords:** aerodigestive tract, radiographic imaging, accidental ingestion, computerised tomography, migratory foreign body

## Abstract

Accidental ingestion of foreign bodies forms a major part of otorhinolaryngological emergencies. It is dangerous, as the foreign bodies tend to perforate the aerodigestive tract. Since endoscopy is diagnostic as well as therapeutic, it is preferred over computed tomography (CT) scan, especially in developing countries.

We present a case of a middle-aged man who presented with fever, neck swelling, and a five-day history of accidental ingestion of a foreign body. He underwent upper gastrointestinal endoscopy, which came out as normal. CT) imaging was performed, which showed migration of the foreign body to the parapharyngeal space and resulting abscess formation. The abscess was drained, and the foreign body was removed transcervically. We discuss this case to stress the role of CT imaging in the diagnosis of foreign bodies of the upper aerodigestive tract and propose an algorithm for the management of such cases. Upper gastrointestinal endoscopy alone would be insufficient to diagnose perforating and migrating foreign bodies, which may cause severe complications if left undiagnosed.

## Introduction

Impaction of foreign bodies in the upper digestive tract is a common pathologic condition in otolaryngology. It is particularly common in children, prisoners, and psychiatric patients. Commonly found objects include fish bones, chicken bones, pieces of glass, dental prostheses, coins, and needles. The initial patient assessment is to identify the type of object, its location in the gastrointestinal (GI) tract, the presence of any associated complications, and the presence of any underlying esophageal conditions [1].

Endoscopic examinations, both flexible and rigid, are considered the investigation of choice for both diagnosis and treatment. Radiographic evaluation is helpful to confirm the location of foreign bodies and associated complications before endoscopy. Computed tomography (CT) scan is superior to plain radiograph for the detection of pharyngoesophageal foreign bodies and provides additional crucial information for the management of complicated cases especially related to sharp or pointed ingested foreign bodies [2,3].

Since endoscopy is diagnostic as well as therapeutic, it is preferred over CT scan especially in low-income countries to minimize the costs associated with an extra investigation. We report a case of migrated sharp foreign body, i.e., fish bone, which presented as a parapharyngeal space abscess where it had migrated and not detected in initial endoscopy.

## Case presentation

A 35-year-old male presented with complaints of dysphagia and swelling in the left lower part of the neck for two days. He revealed a history of a suspected fish bone ingestion one week ago followed by pain and discomfort in the throat. Post-ingestion, the patient sought immediate medical attention. X-ray of the neck was performed in the emergency department, which came out to be normal. He underwent endoscopic assessment of the aerodigestive tract under general anesthesia, which included direct laryngoscopy, esophagoscopy, and bronchoscopy. However, no evidence of foreign body or injury in the upper aerodigestive tract was identified. The patient was reassured and sent home the same day.

Five days later, he noticed swelling in the left lower part of the neck, gradually increasing in size associated with fever and chills. In addition, the patient also reported pus discharge from the swelling for two days. The patient did not have any respiratory distress.

**Figure 1:**
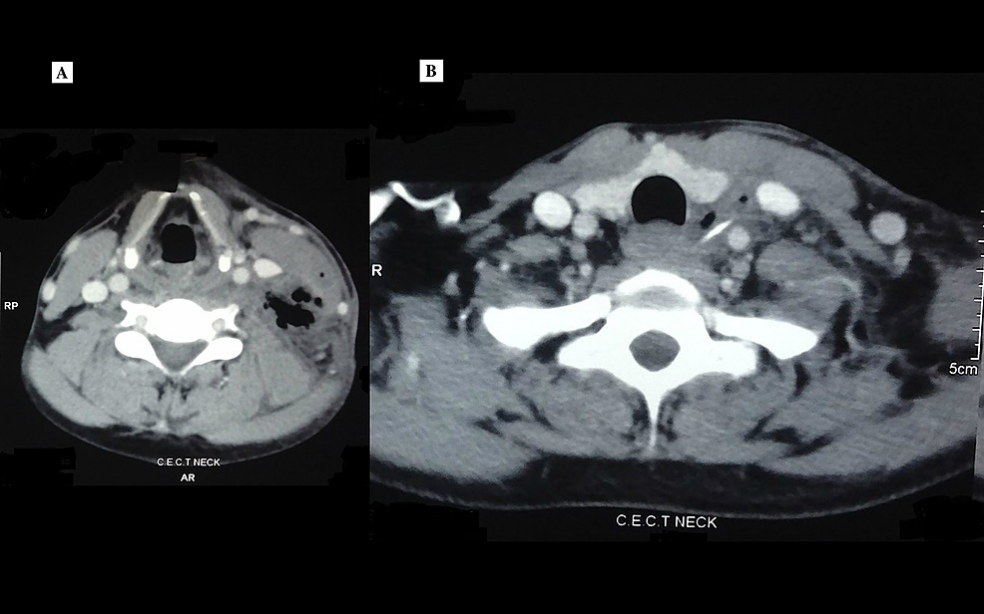
(A) CT showing abscess in the left parapharyngeal space. (B) CT showing the foreign body.

On examination, there was a 3 x 4 cm in size, soft, tender swelling in the left lower part of the neck, with a pus point in the center. The skin over the swelling was erythematous and warm. The remaining examination of the ear, nose, and throat, as well as the general physical examination, were unremarkable. Contrast-enhanced CT was performed from the base of the skull to the fourth thoracic vertebra in which heterogeneously enhancing soft tissue density with multiple air shadows were seen in the left parapharyngeal space (Figure 1A). In the middle of the soft tissue density, between the left internal jugular vein and left carotid artery, a long and narrow calcified lesion was seen, suspected to be a fish bone (Figure 1B).

**Figure 2:**
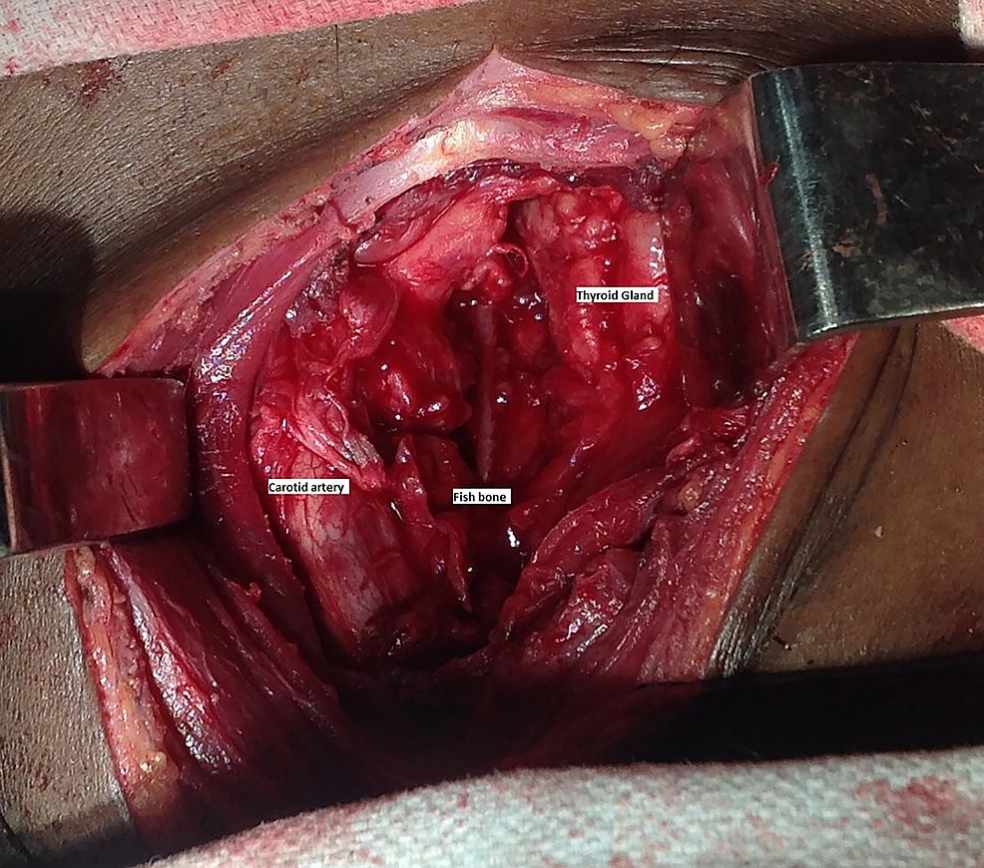
Neck exploration showing the foreign body.

**Figure 3:**
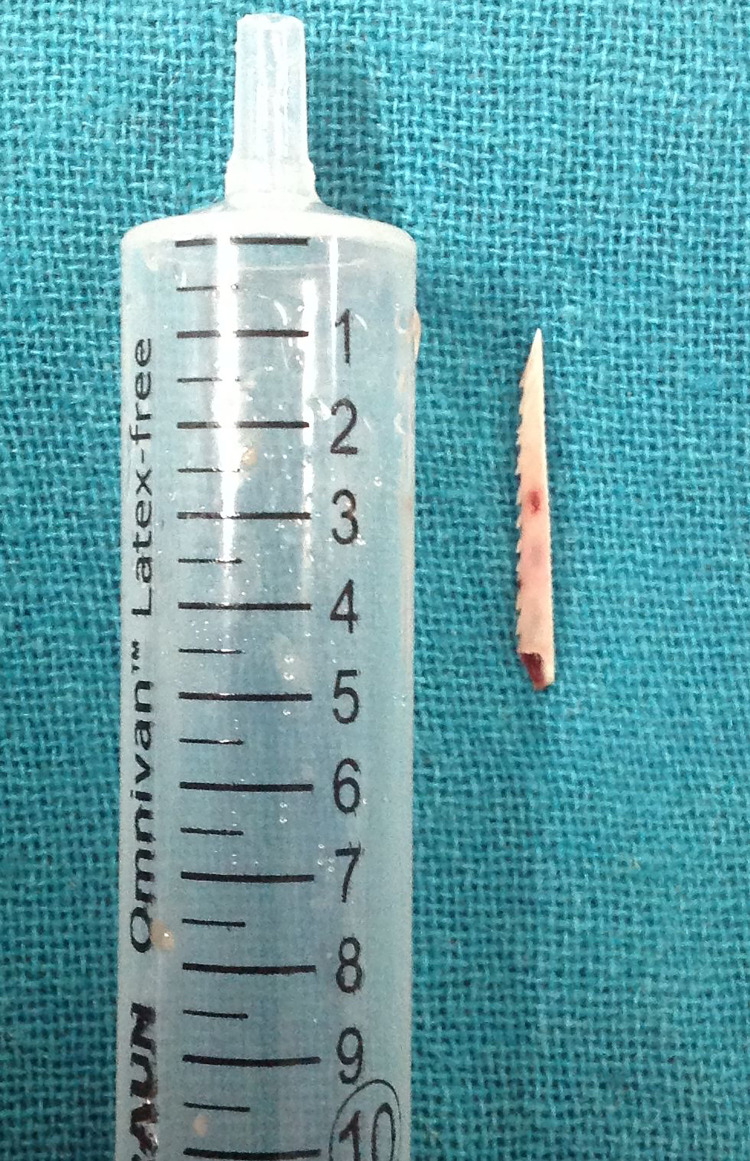
Fish bone.

Furthermore, the patient was taken to the theatre for exploration of the neck under general anesthesia. A vertical incision was made along the anterior border of the left sternocleidomastoid muscle. On entering the plane deep to the sternocleidomastoid muscle, approximately 20 mL of pus was drained out. The carotid sheath was identified, which was swollen and surrounded by extensive granulation tissues. On careful dissection of the granulation tissues, the fish bone was found adjacent to the thyroid lobe (Figure 2). The outer wall of the esophagus was examined, which was unremarkable. The fish bone was removed and was serrated and 4 cm in length (Figure 3). The wound was left open for healing by secondary intention. Post-operatively, the patient was feeling better and the fever settled down. Initially, the patient was kept nil by mouth. To confirm the patency of the tract, repeat endoscopy and barium meal were performed, which showed no leak. Hence, the patient was discharged with oral antibiotics for two weeks as per hospital protocol. No further follow-up was required.

## Discussion

Complications associated with foreign bodies are rare; however, if migrated in the aerodigestive tract, they can cause significant morbidity and, in some cases, mortality [4,5]. The majority of foreign bodies ingested pass through the digestive tract uneventfully. Alleged fish bone ingestion is a potential surgical emergency. Unlike other foreign bodies, they are sharp and have a propensity to perforate the aerodigestive tract. Moreover, they are not inert and often harbor organisms, which easily predispose them to abscess formation. The diagnosis of such patients is also troublesome as almost all patients complain of throat pain due to mucosal aberrations produced by the sharp fish bone, which causes them to be overlooked easily.

Abscess formation following foreign body ingestion is a very rare complication and is usually associated with neglected foreign bodies. In a series of 5,848 patients, Lam et al. reported only five cases of abscess [6]. Perforating and migrating foreign bodies is much rarer [7]. In 10 years, Al Sebeih et al. could only detect 11 patients in which the foreign body perforated the digestive tract and migrated to the neck space [8]. The incidence of neck abscesses following foreign body ingestion has been assessed to be around 0.21% and 0.96% in two separate studies [6-9]. A case series demonstrated that esophageal perforation may occur within 24 hours of ingestion, whereas neck abscesses following ingestion may present after four or more days [10].

The most common site at which a foreign body could perforate the esophagus to become extra-luminal is the oropharynx, which is the narrowest part of the esophagus [6]. X-ray of the neck can easily miss the thin fish bone due to their low sensitivity. An X-ray lateral view of the soft tissue of the neck is usually the first line of investigation followed by a check endoscopy, which is the definitive investigation of choice. The role of CT scans in such cases is debatable. CT scan is not considered cost-effective in many scenarios. However, penetration and migration of the foreign body is a known condition, which is often overlooked in X-ray or endoscopy and leads to complications [11].

Complications depend on the type of foreign body such as button batteries have a propensity to cause edema and ulceration at the region of foreign body impaction due to strong alkali leakage, possibly resulting in esophageal perforation, pneumothorax, or spondylodiscitis [12]. Other complications include periesophagitis, periesophageal abscess, mediastinitis, and upper GI hemorrhage [13]. The most feared complications include aorto-esophageal, subclavian-esophageal fistula, and carotid rupture [14].

Lue et al. have reported sensitivity and specificity of 39% and 72%, respectively, for their plain radiographs [10]. Further Sundgren et al. concluded that radiologic examination adds no advantage in the detection of fish bones and only delays the inevitable endoscopy [15]. CT scan is not considered as the first-line investigation of foreign body ingestion. This may be because of the artifact effect of metallic foreign bodies.

However, in special circumstances such as an absence of intraluminal foreign body as in perforation and migration, endoscopy cannot be helpful. Palme et al. compared plain radiology to CT scan and concluded that CT scan is superior to plain radiography [16]. However, performing a CT scan in all cases of foreign body neck would be in yielding and non-economical.

**Figure 4:**
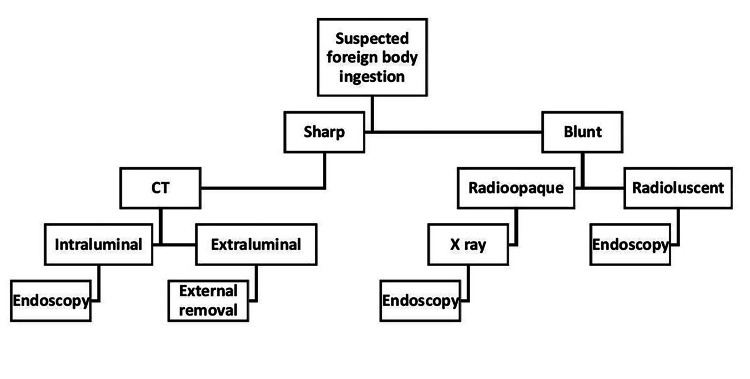
Proposed algorithm for accidental ingestion of foreign body

Hence, we highly recommend CT scans in symptomatic patients with a history of ingestion of sharp radio-opaque foreign bodies such a fish bone or metallic objects. In addition, normal endoscopy and normal X-ray in such patients indicate high suspicion of perforation and migration. Our proposed algorithm is given in Figure 4.

## Conclusions

Foreign body ingestion is a very common situation in otorhinolaryngological emergencies. An early decision of an appropriate investigation can be helpful in rare instances such as perforation and the migration of foreign bodies, which can further lead to some serious and life-threatening consequences. With an example of this presented case, we conclude that performing a CT scan before endoscopy would help to exactly delineate the position (both intraluminal and extraluminal) unlike endoscopy (intraluminal only) and level of impaction or perforation and guide us in the endoscopy.
